# Supplementary material for: Quantum Light in Curved Low Dimensional Hexagonal Boron Nitride Systems
Source: Sci Rep. 2017 Nov 7;7:14758. doi: 10.1038/s41598-017-15398-2 (PMC5676806; doi:10.1038/s41598-017-15398-2)
Supplement: Supplementary file 1 — Supplementary Information [file 41598_2017_15398_MOESM1_ESM.pdf]

# Quantum Light in Curved Low Dimensional Hexagonal Boron Nitride Systems

*Nathan Chejanovsky [1,2], Youngwook Kim [2], Andrea Zappe [1], Benjamin Stuhlhofer [2], Takashi Taniguchi [3], Kenji Watanabe [3], Durga Dasari [1,2], Amit Finkler* [1], Jurgen H. Smet [2] and Jörg Wrachtrup [1,2]*

[1] 3. Physikalisches Institut, Universität Stuttgart, Pfaffenwaldring 57, 70569 Stuttgart, Germany

[2] Max Planck Institute for Solid State Research, Heisenbergstr. 1, 70569 Stuttgart, Germany

[3] National Institute for Materials Science, 1-1 Namiki, Tsukuba, 305-0044, Japan

## Supporting information

### S1 - Bulk BNNT

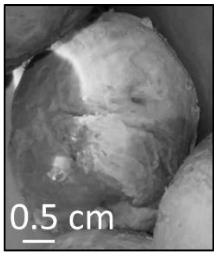


**Figure S1.** An optical picture of 'Bulk' BNNT.

## S2 - Raman analysis of Figure 1a

| Material | | Hexagonal BN (h-BN) | BN nanotubes (BNNT) | cubic BN (c-BN) | Boric acid (B-OH) |
|---|---|---|---|---|---|
| (In-plane) Modes | Diameter [nm] | - | 0.7 - 2.6 | - | - |
| | Raman Species | $E_{2g}$ | $E_{2g}$ | - | $E_{2g}$ |
| | Wavenumber [cm$^{-1}$] | LO: 1453.3 -1614.8, $TO_{\|\|}$: 1364.5- 1453.3 | 1356 - 1380 | TO: 1055, LO: 1304 | 3251 |
| | Energy [meV] | LO: 180 - 200 , $TO_{\|\|}$: 169 - 180 | 169.3 - 171.2 | TO: 130.6, LO: 161.5 | 403.3 |
| (Radial/ Out of plane) Modes | Diameter [nm] | - | 0.2 to 2 | - | - |
| | Raman Species | | Radial breathing modes (RBM) | - | $E_{1g}$ |
| | Wavenumber [cm$^{-1}$] | 729.1- 831.6 | 1000 - 200 | - | 735 |
| | Energy [meV] | $TO_{\perp}$: 103 - 90.3 | 124.1 - 24.8 | - | 91.2 |
| Reference | | 1,2 | 3, 4 | 5 | 6 |

**Table 1.** Raman modes and optical phonon modes for four boron species: h-BN, BNNT, c-BN and boric acid (for which only the B-OH mode is displayed). Modes are generally characterized as in-plane and out-of-plane. This classification is only valid for h-BN, BNNT and B-OH. For c-BN, whose symmetry is zincblende, the transverse and longitudinal optical phonons (TO/LO, respectively) are classified for convenience as in-plane. Each mode's Raman species (if named) is specified, as well as the wavenumber and energy. Diameter is valid only for BNNT whose modes depend on them. For h-BN, the flake thickness affects the modes as well, but for convenience it is not specified here.

It is seen that the Raman peak starts to rise at ~ 1000 cm$^{-1}$. Raman-Stokes modes in BNNTs can be more complex than in 2D h-BN: in BNNTs the diameter as well as the number of walls of the nanotube determine the Raman-Stokes shift. The $E_{2g}$ modes of BNNT are analogous to transverse optical parallel phonons ($TO_{||}$) and longitudinal optical phonons (LO) modes of h-BN. In the limit of increasing tube diameter the Raman shift behavior approached that of 2D h-BN. [3] Nevertheless the broad features of this peak hint to more complex features, other than in-plane modes. For

clarification, in Table 1 we list the known Raman shifts (in wavenumber and energy) and optical phonons for four species of the boron family: h-BN, BNNT, cubic BN (c-BN) and Boric acid. c-BN nitride is an $sp^3$ allotrope of BN which is isoelectric to diamond. [7,8] The radial modes of BNNT would fit only the start of the rise of the main peak at ~ 1000 $cm^{-1}$. [3] Tube bundling mode-softening is well known from carbon nanotube research. However, *ab initio* calculations have shown that softening is only on the scale of ~ 10 $cm^{-1}$ for all modes in BNNT. [4] Since the BNNT tube diameters we use are in the range of 3 nm to 6 nm [9] and BNNT radial modes are for diameters smaller than 2 nm, we discard them as the cause of the broadening of this peak. Due to contributions in the wavenumber range of ~1000 $cm^{-1}$ to 1379 $cm^{-1}$ we conclude that some $sp^3$ bonds (as attributed for higher energy excitations) [10] present in our BNNT material could give Raman modes similar to c-BN modes (see Table 1). Similar c-BN modes have been attributed to $sp^3$ hybridization of 2D h-BN at grain boundaries using high-resolution electron energy loss spectroscopy. [2] Additionally, a peak at ~ 3200 $cm^{-1}$ is visible, which can correspond to B-OH/B-O bonds [6,11], or possibly to moisture. [11,12] For clarity, the modes of the various BN species are displayed on the wavenumber axis.

**S3 - BNNT Exfoliation**

We also attempted to exfoliate BNNT material, similar to the methods used for 2D h-BN. However, emitters bleached after ~ 1 hours of stable excitation. These samples were prepared using the same methods as h-BN exfoliated in Ref. 13 , with the difference that at the end of the exfoliation procedure they were submerged for 10 minutes in ethyl alcohol and afterwards dried in $N_2$ gas.

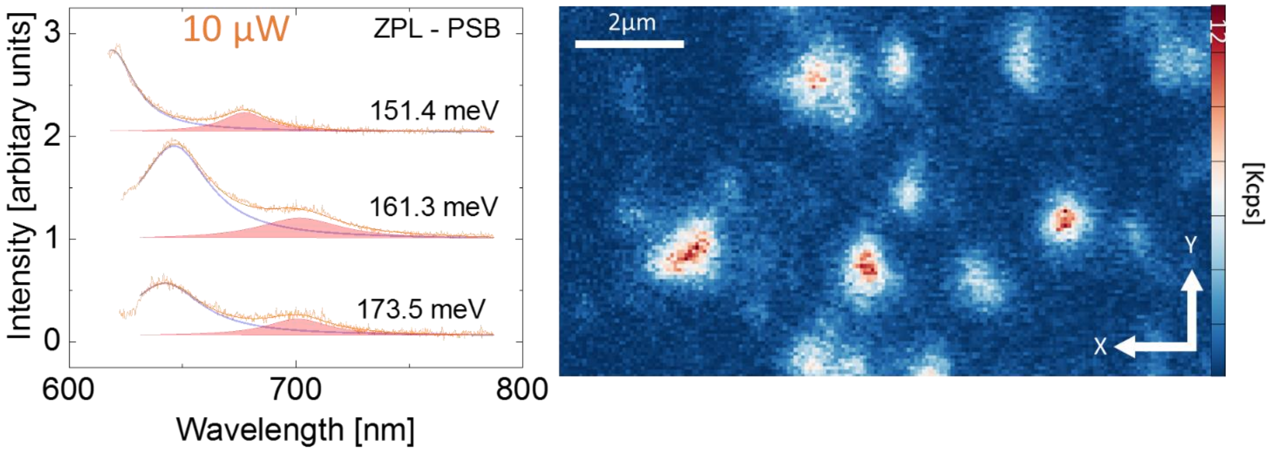


**Figure S2.** PL spectra of exfoliated BNNT with the corresponding confocal scan.

## S4 – Peak analysis and detuning of Figure 2a1

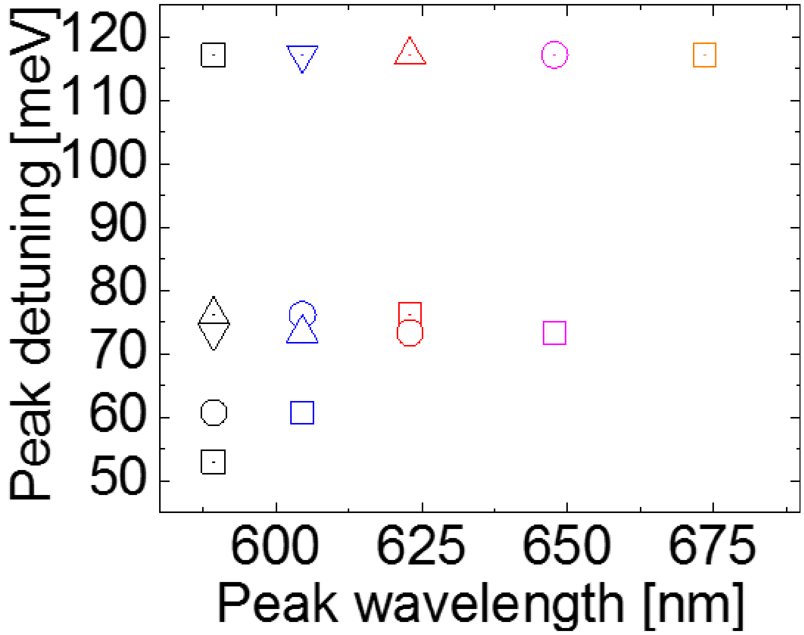


**Figure S3.** detuning of the five spectral peaks to the last spectral peaks in energy.

For the PL spectra in Fig. 2a1, peaks were fitted individually with Lorentzian/Gaussian functions. These were then renormalized to portray the peak they represent on the original PL spectra. We denote the first peak as a potential ZPL, whereas the remaining five as potential PSBs. Using this classification we subtract the energy between adjacent peaks. This is seen in Fig. S3. Using Table 1, the energy detuning is closest to the range of the Radial Breathing Modes (RBM) of BNNT. In some instances we observed that when switching to 594 nm excitation on the same emitter, the emission spectrum is not seen. This leads us to believe the first peak, at ~ 589 nm, is the ZPL, which can be shifted by the local electric environment.

**S5 - SQE photo-dynamics Analysis for Figure 2 in main text**

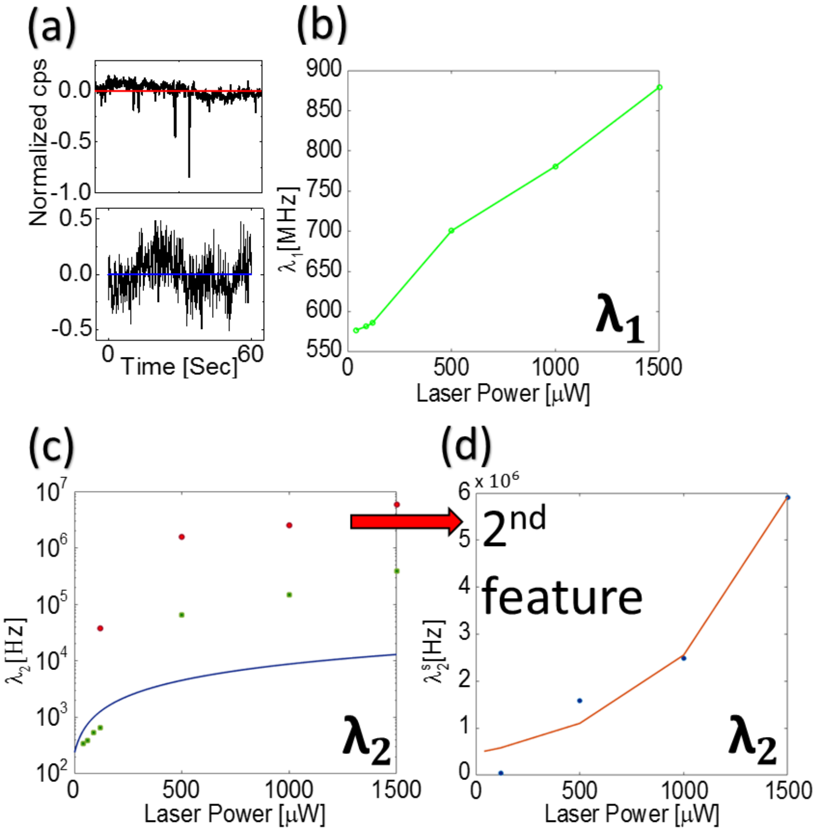

**Figure S4.** (a) Normalized emission photon trace where the average emission is set as zero for two both powers. For low power (blue curve) emission is uniformly distributed around the average whereas for high power (red curve) deviations from the average (down shifts) with different magnitudes are seen. (b) excited state frequencies as a function of 532 nm CW excitation power. (c) metastable state frequencies as a function of 532 nm CW excitation power in logarithmic scale (green points and blue fitting). Red points are the frequencies of the intermittency occurring at higher excitation powers. (d) Best function fit for intermittency in (c).

In Figure 2d (main text) for low excitation power (40 μW) only one time decay exponent can be fitted, whereas for high power (500 μW) a shorter decaying exponent is seen. To gain insight, in Figure S4a we plot the normalized emission photon trace by setting the average emission as zero for two powers (low/high) for 60 seconds (Fig. S4a bottom/top, respectively). The average emission traces are the blue/red curves, respectively. For the low power trace the emission is dispersed evenly ~ 50% below and above the average emission. However, for the high power trace we see bursts of down-shifted emission well below the distribution of emission around the average. We note that these down shifts do not have the same percentage and we can roughly classify ~ 20%, 45% and 84% shifts. This indicates a switch of the emitter to a dark state which can occur on a time scale faster than our photon detection whose duration can vary, thus the different down shift percentage. Therefore, the emitter can be trapped in a dark state due to an intermittent blink to trap states or a different emitter charge state. Using this interpretation, we can now attribute the short decaying component to the switch of the emitter to the dark state, whose duration can vary and does not always register fully on our time photon trace. Therefore, our photodynamic analysis is for a three state system similar to that depicted in Fig. 1g – inset. The second order correlation describing the photo-physics of our emitter can be well described using:

$$g^{(2)}(\tau) = 1 - (1+a)e^{-\frac{t}{\tau_1}} + ae^{-\frac{t}{\tau_2}}$$

where the parameters $a, \tau_1, \tau_2$ are power-dependent. The parameters are derivable from a three-level model which better explains the observed experimental data. The three-level system consists of a ground, excited, metastable state and an additional high-lying state to which the metastable is coupled to optically. [14] To find the transition rates of the model $k_{ij,}$ we follow the approach applied to describe the photophysics of the silicon-vacancy color center in diamond. [14] These frequency rates are shown in Fig. S4c, on a logarithmic scale for $\lambda_2$ for both components. Fig. S4b displays the $\lambda_1$ frequency. When the power exceeds 120 μW a high frequency (MHz) component is seen (red dot – red curve added for emphasis) larger by a few orders of magnitude than the original decay frequency (black dots). The limiting values (i.e., $P \to 0, P \to \infty\ (2000\mu W)$) of various parameters that go into the analysis are:

$$\tau_1^0 = 1.75\text{ ns}, \tau_2^0 = 4.175\text{ ms}, \tau_2^\infty = 1\ \mu\text{s}, a^\infty = 0.16$$

**S6 - PL spectra asymmetric fitting functions**

The following function was used, where $f(x)$ denotes the normalized PL intensity and x denotes the wavelength:

$$f(x) = y_0 + A\left(\frac{1}{1+e^{\frac{-\left(x-x_c+\frac{w_1}{2}\right)}{w_2}}}\right)\left(1-\frac{1}{1+e^{\frac{-\left(x-x_c-\frac{w_1}{2}\right)}{w_3}}}\right)$$

**S7 - 2D h-BN on diamond pillars**

h-BN was exfoliated on a $Si/SiO_2$ substrate for maximum optical contrast. Using a PDMS stamp transfer method [15] the flakes were then transferred to the diamond pillars. The samples were

annealed at 500 $^oC$ in an Ar/H environment for 1 hour, which should not damage the h-BN layers. [16] An AFM scan is displayed below in Fig. S5c, with the corresponding heights for each line (1) and (2) (Fig. S5a and Fig. S5b, respectively).

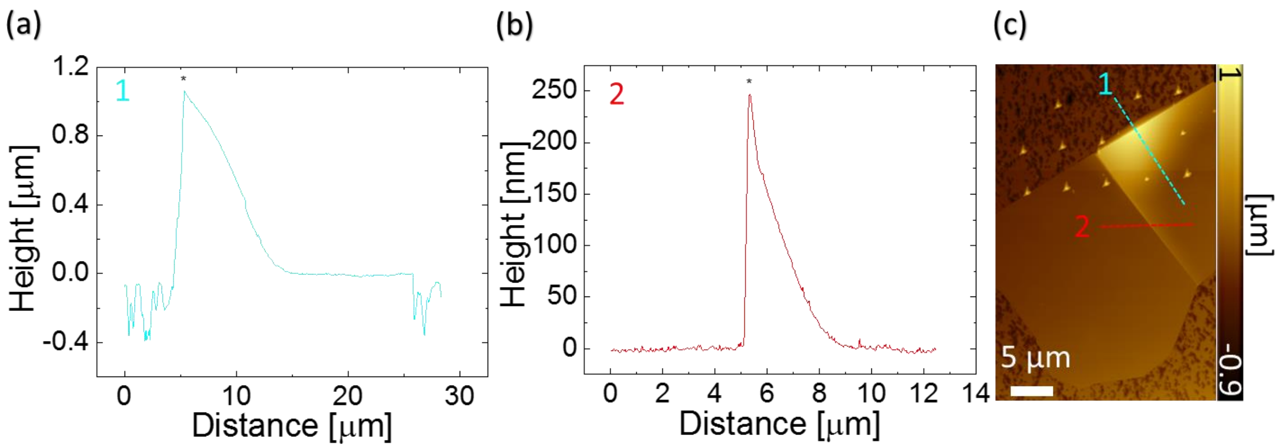


**Figure S5.** AFM topography curves for area 1 (a) and for area 2 (b) marked on the AFM map (c) for pillar '1' in the main text and for the height for the are marked with 'Fold' in the main text.

The annealing procedure used removes organic contaminants and does not introduce pinholes for temperatures below 600 $^oC$. [16]

**S8 – Example of absorbed PSB for suspended material**

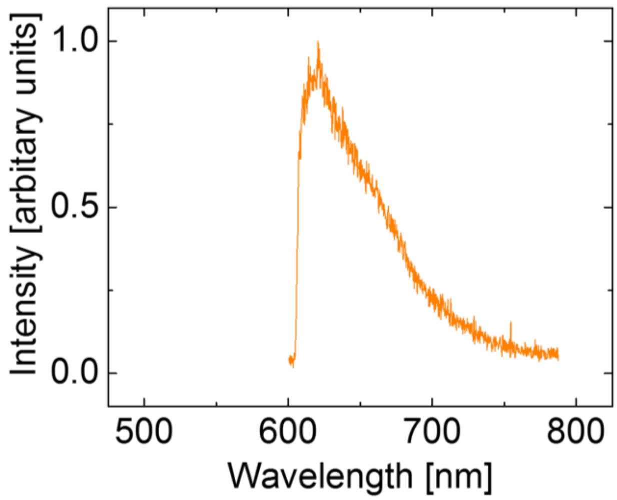


**Figure S6.** PL spectra for a suspended emitter using 594 nm excitation.

**S9 – Curved 2D h-BN on $ZrO_2$ hemisphere**

A hemisphere of cubic $ZrO_2$ with a diameter of 3 mm (A.W.I. Industries Inc.) was cleaned in the same method as described as cleaning $SiO_2$ for the BNNT oxygen plasma method (piranha + plasma). The half sphere was then fixated to a clean $SiO_2$ substrate using glue. Single crystal h-BN was then exfoliated on the sphere (as depicted in figure S7.a). Thus ensuring that adhesion to surface would be imperfect – creating curvature in the flake for the areas not in contact with the hemisphere. A large flake containing curved regions was located using a wide field micro scope (Figure S7.b and S7.c – with different focus regions due to the substrate curvature, Inset Figure S7.b – curved region, where red arrow is for emphasis of the curved flake area).PL measurements of this flake were conducted in two modes using 12μw 594nm excitation: The first mode is directly after exfoliation. The second mode is after cleaning the sphere in acetone at 80 C° for 15 minutes, 15 minutes of room temperature ethyl alcohol and 15 minutes of minutes of room temperature isopropyl alcohol followed by drying the half sphere using pure $N_2$ gas, similar methods used for preparing the h-BN material in Ref. [17] As we will show, this resulted in different results.

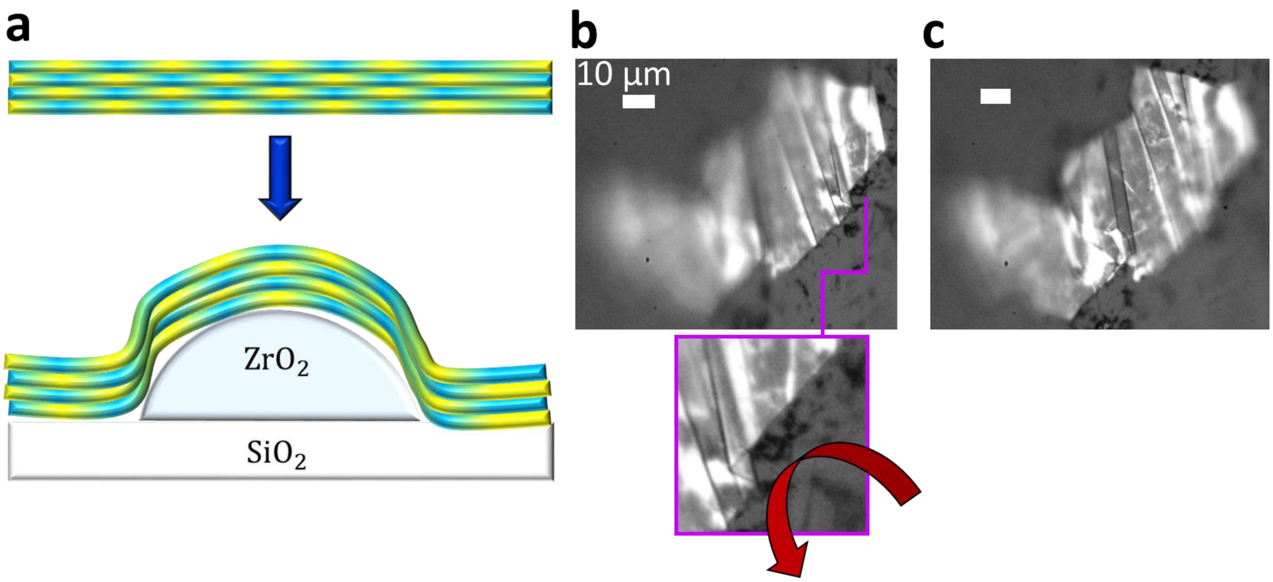


**Figure S7. (**a) Illustraion of sample preparation scheme. (b),(c) microscope wide field images of large exfoliated flake on the half sphere. Inset displays curved areas in detail.

**S10 – PL mode 1 – directly after exfoliation**

We probe a large area of the flake which in the reflection scan (nw power with no filters) shows multiple curved areas (Figure S8.a – indicated by the red curved arrow). A 600LP filtered scan reveals (Figure S8.b, X/Y axis and X/Z axis) ***almost no photoluminescence*** except for one region (Figure S8. C). This tendency of few (perhaps one or two) emitting areas in a ~ 40 x 40 μm scan was consistent throughout this flake. The PL spectra for two segments denoted D1 and D2 is displayed in Figure S8. D. D1 has broad spectral features whereas D2 has sharp features. We tentatively assign the features in D2 to sharp emitters of Type '2' as described in ref. [18], or possibly to defect energy states of the $ZrO_2$ hemisphere, due to the consistency of these sharp peaks to appear always at ~ 692 nm with no detuning, whereas detuning is typical for emitters in h-BN. However, these were the only areas showing emission in this segment of the flake. Emission from

these were completely stable with no sign of bleaching or blinking. If we assume these originate from the h-BN, we can possibly attribute the stability to a the $ZrO_2$ hemisphere substrate, presumably similar to the stabilizing effect assigned in ref. [18] to $Al_2O_3$.

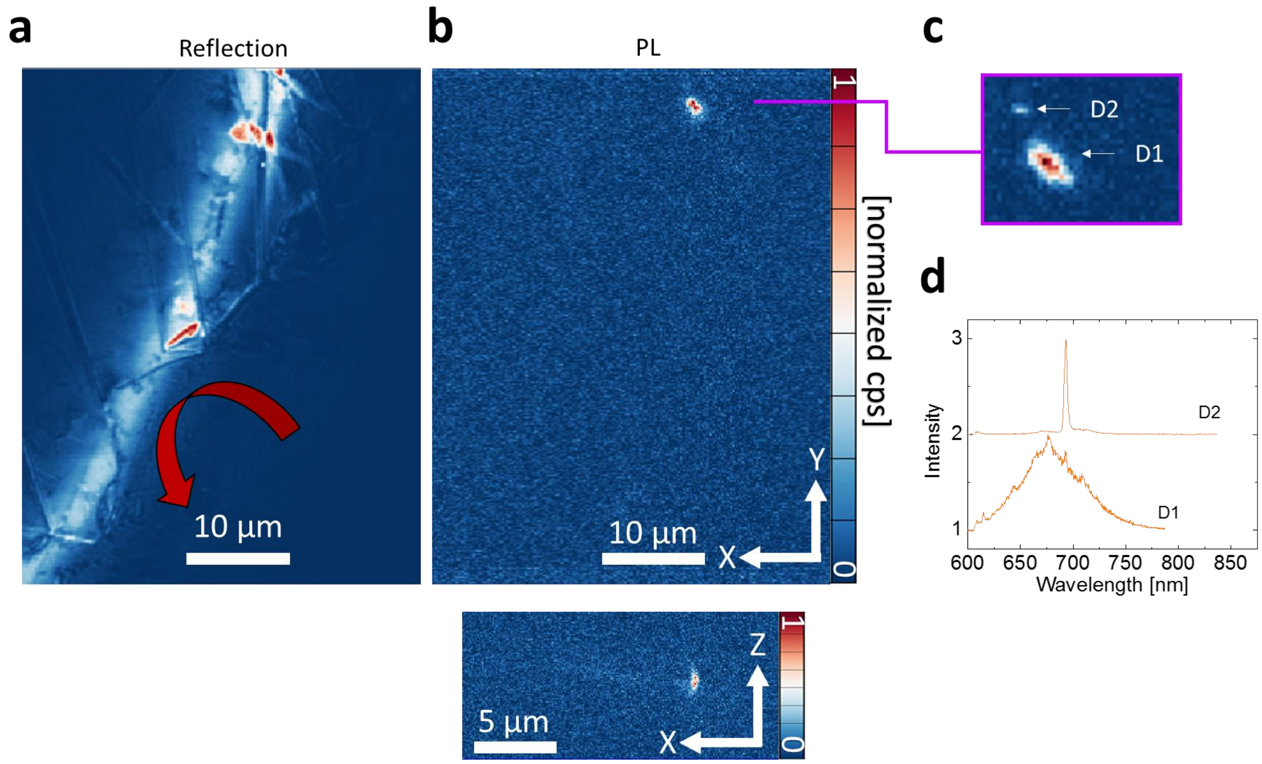


**Figure S8.** Reflection / PL confocal scan of a ~ 40 x 40 micron scan range (a,b, respectively). (c) enlarged PL segment for only area where emission is seen. d. PL spectra for both area named D1 and D2 in c.

## S11 – PL mode 2 – after exposure to solvents

The picture of isolated (if any) emitting segments in S10 changed drastically after the exposure to solvents. Figure S9.a / b displays the reflection / PL (respectively) of a cross section area inside a flake after solvent exposure. We note that due to the non-straight topography of the flake our z focus point along the x axis for the PL emission is limited. This limited focus area is depicted in Figure S9.b. The reflection image does not suffer from this limitation due to the high brightness. The reflection image reveals in detail wrinkled lines are on the flake. Red arrows mark the

pronounced curved regions of the flakes. Clustering of isolated emission point along these lines are visible (Figure S9. B). These emission points exhibited blinking behavior, a time trace of 200 seconds of such behavior is depicted in Figure S9.c  We mark three of these as D3,D4 and D5. Figure S9.d displays the PL spectra. The PL spectra resembles that seen in Figures 1.f, 4 in the main text. We note that these spectral features **were not** seen prior to solvent exposure. Due to the blinking nature of the emitters, a clear anti-bunching signal is difficult to observe, nevertheless for emitter D4 an indication of anti-bunching could be seen (Figure S9. e). The other emission points which were not along the wrinkled lines were unstable and immediately bleached. The correlation of the emission areas with the h-BN flake structure and lack of almost any emission of the material prior to solution exposure is a strong indicator of a reaction of the flake with the seemingly benign solvents. Due to the non-invasive nature of the solvents, we can assume that the interaction is on the surface area of the flake, possibly oxide groups attach to the flake exterior favorable at defective sites. These result can indicate that emitters seen in commercial h-BN dispersed in ethanol/water can originate from the reaction of h-BN with liquids. Thus defective sites more prevalent in monolayer 2D h-BN would be reactive in such a solution.

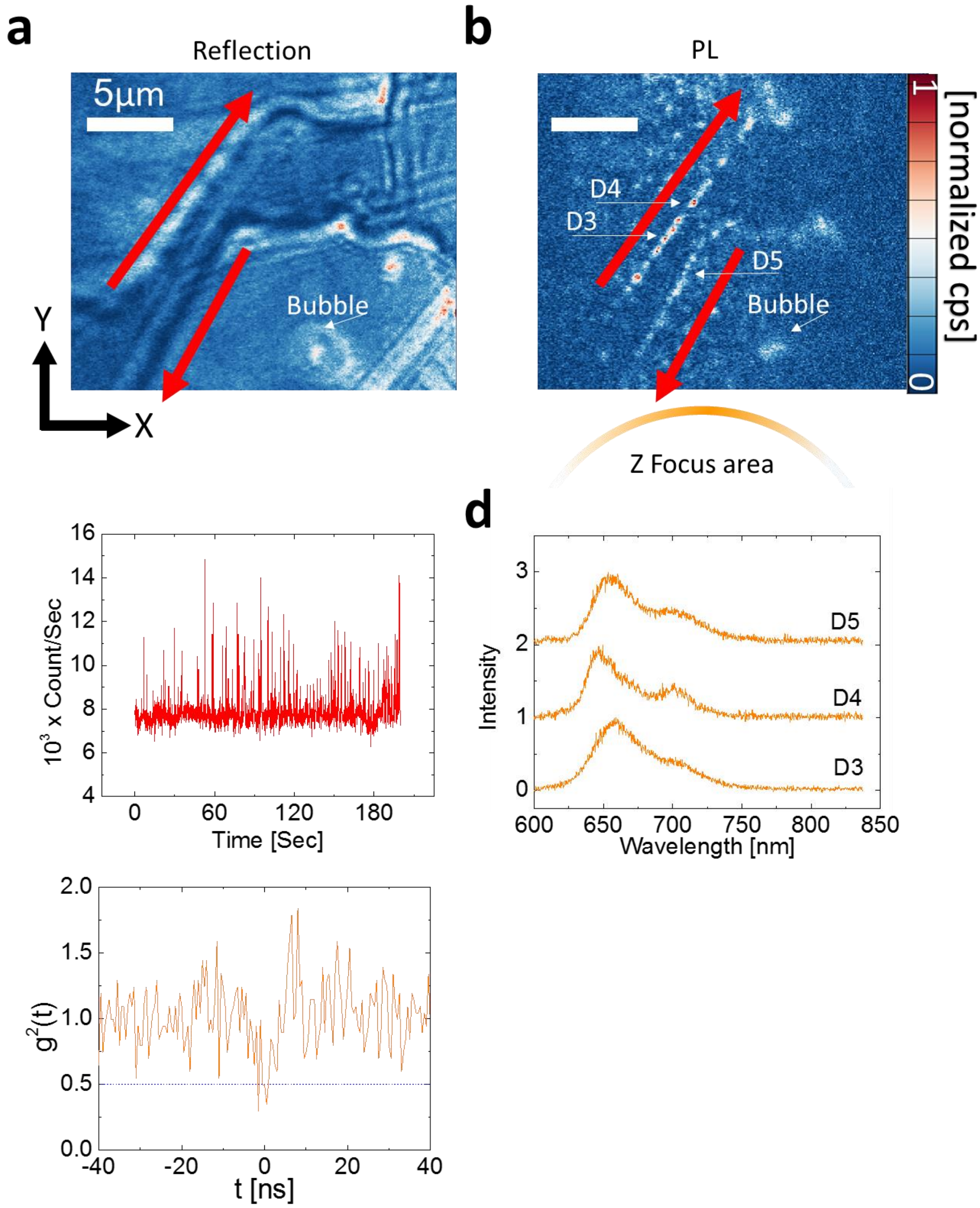


**Figure S9.** (a) Reflection PL inside a flake region, pronounced wrinkled lines are marked with red arrows. (b) Filtered PL of the same region. Isolated emission points are marked with D3,D4 and D5. The z axis focus area visible on the x axis is marked with a half orange circle. (c) Emission time trace for one

emitter –exhibiting blinking behavior. d. PL spectra for D3,D4 and D5. e. Auto-correlation measurement for D4.

## S11 – Power-dependent photo-dynamics for Figure 1c

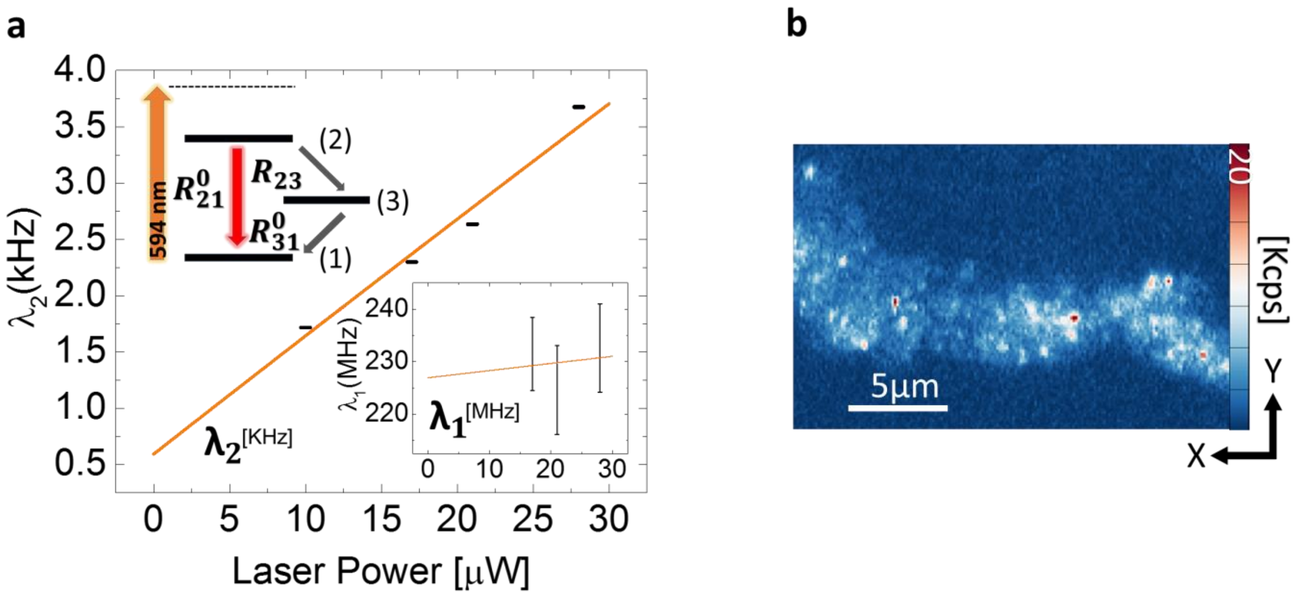


**Figure S10.** (a) Metastable state frequencies; Inset: excited state frequencies. Illustration depicts three state energy level scheme. (b) Optical scan using 22 μW 594 nm CW excitation reveals possible isolation of single QEs for BNNT suspended on Al needles.


REFERENCES:

1. Serrano, J. *et al.* Vibrational properties of hexagonal boron nitride: Inelastic X-ray scattering and Ab Initio calculations. *Phys. Rev. Lett.* **98,** 12–15 (2007).

2. Nai, C. T., Lu, J., Zhang, K. & Loh, K. P. Studying Edge Defects of Hexagonal Boron Nitride Using High Resolution Electron Energy Loss Spectroscopy. *J. Phys. Chem. Lett.* **6,** 4189–4193 (2015).

3. Arenal, R. *et al.* Raman Spectroscopy of Single-Walled Boron Nitride Nanotubes. *Nano Lett.* **6,** 1812–1816 (2006).

4. Ludger, W., Rubio, A., de la Concha, R. & Loiseau, A. Ab initio calculations of the lattice dynamics of boron nitride nanotubes. *Phys. Rev. B* **68,** 045425 (2003).

5. Reich, S. *et al.* Resonant Raman scattering in cubic and hexagonal boron nitride. *Phys. Rev. B* **71,** 205201 (2005).

6. Krishnan, K. The Raman spectrum of boric acid. *Proc. Indian Acad. Sci.* **60,** 103–109 (1963).

7. Schmid, H. K. Phase Identification in Carbon and BN Systems by EELS. *Microsc. Microanal. Microstruct.* **6,** 99–111 (1995).

8. Karch, K. & Bechstedt, F. Ab initio lattice dynamics of BN and AlN: Covalent versus ionic forces. *Phys. Rev. B* **56,** 7404–7415 (1997).

9. Tiano, A. L. *et al.* Boron nitride nanotube: synthesis and applications. *Proc. SPIE* **9060,** 906006–906019 (2014).

10. Museur, L. & Kanaev, A. Near band-gap electronics properties and luminescence mechanisms of boron nitride nanotubes. *J. Appl. Phys.* **118,** 084305 (2015).

11. Tiano, A. *et al.* Thermodynamic Approach to Boron Nitride Nanotube Solubility and Dispersion. *Nanoscale* 4348–4359 (2016). doi:10.1039/C5NR08259E

12. Nautiyal, P. *et al.* Oxidative Unzipping and Transformation of High Aspect Ratio Boron Nitride Nanotubes into White Graphene Oxide Platelets. *Sci. Rep.* **6,** 29498 (2016).

13. Chejanovsky, N. *et al.* Structural Attributes and Photodynamics of Visible Spectrum Quantum Emitters in Hexagonal Boron Nitride. *Nano Lett.* **16,** 7037–7045 (2016).

14. Neu, E., Agio, M. & Becher, C. Photophysics of single silicon vacancy centers in diamond: implications for single photon emission. *Opt. Express* **20,** 19956–19971 (2012).

15. L., W. *et al.* One-Dimensional Electrical Contact To a Two-Dimensional Material. *Science (80-. ).* **342,** 614–618 (2013).

16. Li, L. H., Cervenka, J., Watanabe, K., Taniguchi, T. & Chen, Y. Strong oxidation resistance of atomically thin boron nitride nanosheets. *ACS Nano* **8,** 1457–1462 (2014).

17. Tran, T. T., Bray, K., Ford, M. J., Toth, M. & Aharonovich, I. Quantum Emission From Hexagonal Boron Nitride Monolayers. *Nat. Nanotechnol.* **11,** 37–41 (2016).

18. Li, X. *et al.* Non-Magnetic Quantum Emitters in Boron Nitride with Ultra-Narrow and Sideband-Free Emission Spectra Non-Magnetic Quantum Emitters in Boron Nitride with Ultra- Narrow and Sideband-Free Emission Spectra. *ACS Nano* (2017). doi:10.1021/acsnano.7b00638
